# Inter-observer variability in breast segmentation and its impact on focused ultrasound thermal therapy modeling

**DOI:** 10.1088/1361-6560/ae7cd0

**Published:** 2026-07-02

**Authors:** Benjamin Jackson, Nicole Peterson, Taylor Forbes, Rachel Harris, Chloe K Nelson, Amelia Martin, Brianne Geiger, Jacob Moulder, Jacob Lehnhof, David B Dahl, Allison Payne, Christopher R Dillon

**Affiliations:** 1 Department of Mechanical Engineering, Brigham Young University, Provo, UT, United States of America; 2 Department of Statistics, Brigham Young University, Provo, UT, United States of America; 3 Department of Radiology and Imaging Sciences, University of Utah, Salt Lake City, UT, United States of America

**Keywords:** segmentation, interobserver variability, focused ultrasound, thermal therapies, treatment planning

## Abstract

*Objective*. Simulation-based treatment planning (SBTP) could improve magnetic resonance-guided focused ultrasound (MRgFUS) breast cancer treatments. Medical image segmentation is an essential SBTP step and may introduce uncertainty through interobserver variability. This exploratory study quantifies interobserver variability in breast magnetic resonance imaging (MRI) segmentation and assesses its impact on simulated MRgFUS breast cancer treatment outcomes. *Approach.* Nine researchers segmented three breast MRI. Interobserver variability amongst segmentation datasets was quantified using the surface Dice coefficient (sDICE), modified Hausdorff distance (MHD), and a multi-label, volume-weighted Dice similarity coefficient (wDICE). Segmentations were used in acoustic and thermal simulations to evaluate MRgFUS treatment outcomes including maximum temperature rise, thermal dose volume (TDV), and distance between the target location and simulated thermal center of mass (TCOM). Exploration of which labels and metrics best explained variation in treatment outcomes was performed with multiple regression using the ordinary least squares method and leave-one-out cross validation. *Main results.* Interobserver variability led to substantial differences in simulated outcomes, with maximum temperature changes up to 62.9 °C, TDV differences up to 324 mm^3^, and target-to-TCOM shifts as large as 3.6 mm. Regression analysis identified wDICE as a stable global metric across outcomes, while sDICE and MHD for the biopsy marker label highlighted label-specific variability. These results suggest wDICE as an overall indicator of segmentation variability for MRgFUS SBTP, with MHD and sDICE as potentially useful metrics for identifying labels where reduction of variability would be most impactful. *Significance.* Interobserver variability can meaningfully affect simulated MRgFUS treatment outcomes, warranting further study of clinical interobserver variability for SBTP. The exploratory regression modeling demonstrated here provides a potential methodology for identifying labels where reduction of interobserver variability would be most impactful.

## 1. Introduction

Magnetic resonance-guided focused ultrasound (MRgFUS) is a rapidly developing, non-invasive alternative to traditional therapies for the treatment of many diseases, including breast cancer (Cavallo Marincola *et al* 2015, Zulkifli *et al* 2023). Mechanisms of focused ultrasound include histotripsy, targeted drug delivery, opening of the blood-brain barrier, and thermal ablation (Eranki *et al* 2020, Arsiwala *et al* 2021, Bachu *et al* 2021, Mouratidis *et al* 2021, Xu *et al* 2021, Baek *et al* 2022, Tang *et al* 2023, Zulkifli *et al* 2023, Leporace *et al* 2025). For thermal ablation of breast cancer, MRgFUS directs high-intensity (∼1500 W cm^–2^) acoustic waves to a focus within the tumor. At the focus, acoustic energy is converted to thermal energy, causing rapid heating and necrosis of the tumor (Robertson and Baker 2001, Bachu *et al* 2021). Magnetic resonance imaging (MRI) enables real-time guidance and monitoring of treatment (Schlesinger *et al* 2013, Kuroda 2018, Moonen *et al* 2025). The fully non-invasive nature of MRgFUS can greatly reduce recovery time and side effects for patients relative to open surgery, chemo- and radiation therapy (Elhelf *et al* 2018). The objective of this study is to quantify interobserver variability from breast MRI segmentation in the MRgFUS treatment planning phase, and to determine if simulated MRgFUS breast cancer treatment outcomes are sensitive to this interobserver variability.

Currently, clinicians performing MRgFUS treatments rely on experience, intuition, and magnetic resonance (MR) thermometry to plan and make adjustments that seek to preserve healthy tissues and ensure complete ablation of the tumor (Loeve *et al* 2016, Meng *et al* 2020). This somewhat ad hoc approach is warranted from a safety perspective but is not optimal. Additionally, MRgFUS is hindered by the inability of traditional MR thermometry to accurately measure temperatures in and near adipose tissue, which is extensive in the breast (Josset *et al* 2025). These challenges can result in long treatment times, skin burns, strict exclusion criteria, incomplete sonications, and poor outcomes (Cavallo Marincola *et al* 2015, Payne *et al* 2021, Hyvärinen *et al* 2022) and indicate an opportunity for improved treatment planning in MRgFUS.

Radiotherapy is a well-established non-invasive cancer treatment that consistently uses patient-specific treatment planning to boost dose delivery to cancerous tissues while reducing radiation exposure to healthy organs (Shiradkar *et al* 2016). Radiotherapy treatment planning includes a well-defined process for treatment optimization that includes medical imaging, target volume segmentation, and predictive calculations of radiation dosage (Fiorino *et al* 1998, Shiradkar *et al* 2016, Sanders *et al* 2022). A similar patient-specific simulation-based treatment planning (SBTP) process could benefit MRgFUS (White *et al* 2008, Payne *et al* 2021, Hyvärinen *et al* 2022).

Achieving increased MRgFUS performance through SBTP requires extensive validation of the proposed process, which includes medical imaging, segmentation, acoustic and thermal simulations, and treatment outcome predictions (White *et al* 2008, Paulides *et al* 2013, Gupta *et al* 2024). Several studies have sought to validate acoustic and thermal simulations and their ability to predict thermal behavior of various tissues (Johnson *et al* 2018, Hansen *et al* 2021, Richards *et al* 2024). Studies have also established the sensitivity of transcranial focused ultrasound simulation results to computed-tomography imaging parameters (Montanaro *et al* 2021). Some work has been done exploring different automated segmentation methods for tumor identification in focused ultrasound therapies (Zhang *et al* 2016, Vargas-Olivares *et al* 2019). Relatively little work has been done exploring the influence of segmentation variability on simulated MRgFUS outcomes.

**Figure 1.**
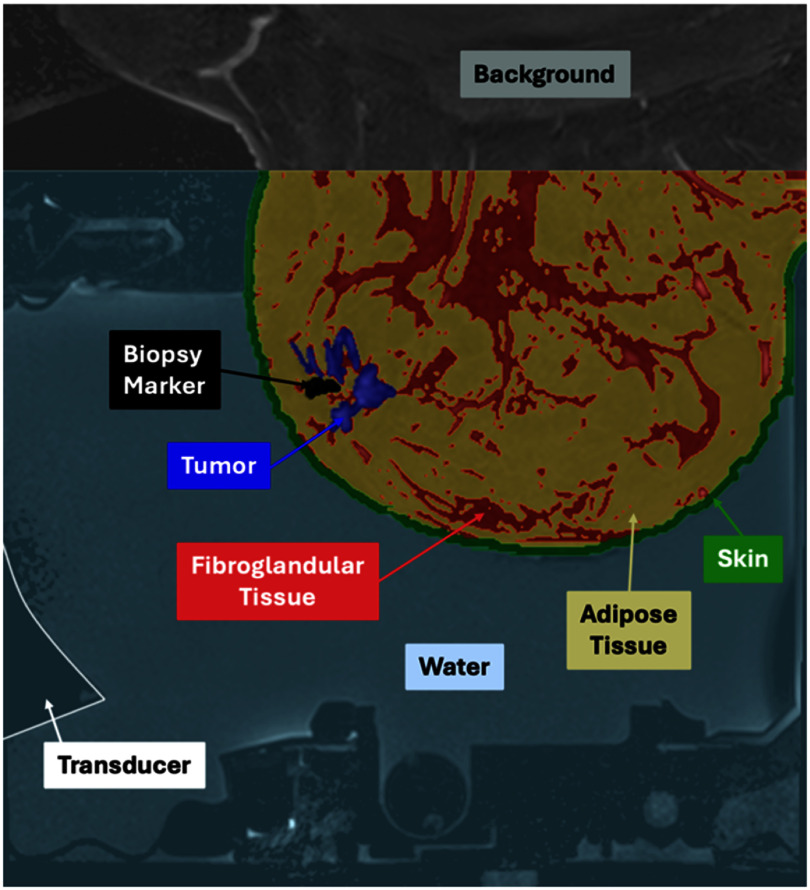
Breast segmentation example overlaid on a T1-weighted, dynamic contrast enhanced MRI of Patient 1.

Segmentation refers to the process of converting medical imaging such as MRI into a 3D model of patient anatomy with each tissue type partitioned and labeled appropriately (see figure 1). The dataset resulting from segmentation is assigned material properties for each tissue and used as an input for other steps within the SBTP process including acoustic and thermal simulations.

There is inherent variation in segmentation. Imprecise tissue boundaries and limited resolution in medical imaging unavoidably result in different tissue partitioning and labeling by different individuals (Fiorino *et al* 1998, Lenfant *et al* 2025). The resulting variation is called interobserver variability. From analysis of segmentation for radiotherapy planning, meaningful interobserver variability was found even when segmentation was performed by expert radiologists (Fiorino *et al* 1998, Vinod *et al* 2016a, Covert *et al* 2022, Sanders *et al* 2022). In those studies, segmentation uncertainty was established as one of the main sources of dosimetry uncertainty. This variability greatly influenced radiotherapy treatment planning outcomes and motivated work to establish metrics and guidelines for analyzing and reducing segmentation variability (Vinod *et al* 2016b, Patrick *et al* 2021, Poel *et al* 2021). The progression of these studies for radiotherapy planning provides a potential roadmap for assessing segmentation variability in MRgFUS therapies.

In this exploratory study, we quantify interobserver variability in breast cancer segmentation datasets and the impact that interobserver variability has on simulated MRgFUS treatment outcomes. Multiple individuals generate segmentation datasets of the same breast MRI scans. Overlap- and distance-based metrics are used to quantify the interobserver variability. Acoustic and thermal simulations are performed with each segmentation dataset. Treatment outcomes are calculated from the simulated temperature distributions and regression analysis is performed to explore the connection between simulated outcome variability and the overlap- and distance-based metrics. By exploring how interobserver variability impacts clinical treatment outcomes, this study provides preliminary evidence regarding the rigor and robustness needed for segmentation in a SBTP pipeline for improved and optimized MRgFUS treatment planning.

## 2. Methods

### 2.1. Medical imaging

**Figure 2.**
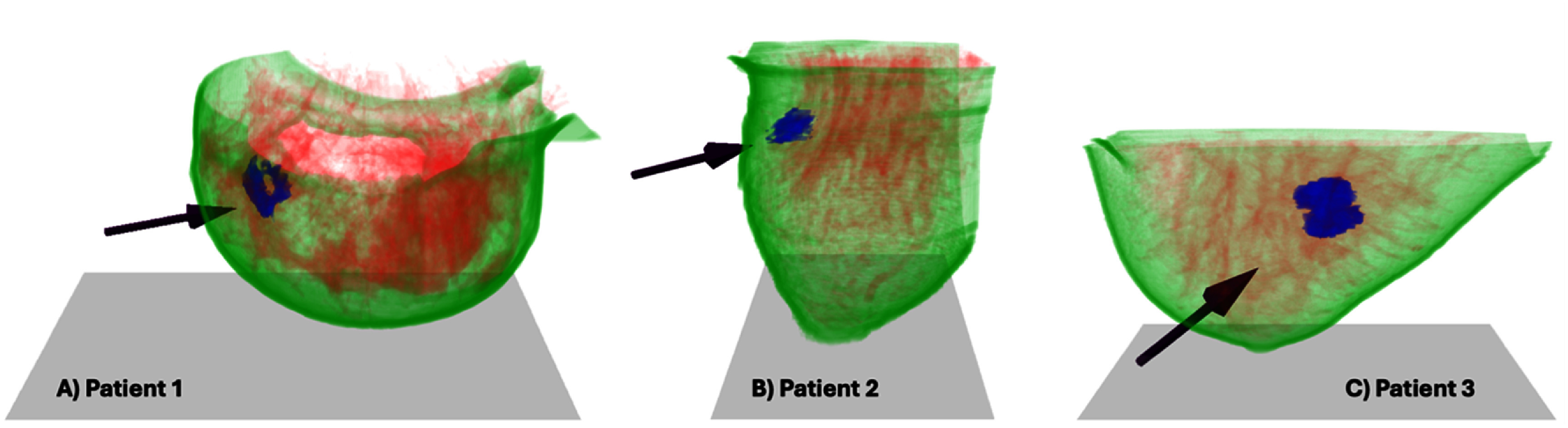
3D-rendering of tumor (blue), fibroglandular tissue (red), and skin (green) in A) Patient 1, B) Patient 2, and C) Patient 3. Arrows indicate the ultrasound propagation direction.

Medical imaging for the study included T1-weighted dynamic contrast enhanced MRI (DCE-MRI) scans of three patients diagnosed with breast cancer (see figure 2).

The first two patients participated in a clinical trial for MRgFUS treatment (NCT05291507) and were imaged on a Siemens 3 T Skyra scanner (3D volume interpolated breath hold exam, repetition time (TR) = 4.19 ms, echo time (TE) = 2.19 ms, flip angle (FA) = 10°, field of view (FOV) = 190 × 280 × 112 mm, 1 mm isometric resolution, fat saturated). All data was anonymized before use in this study in accordance with local institutional review board protocols.

The third scan was acquired from a public medical imaging database of deidentified cancer imaging scans, the cancer imaging archive (Clark *et al* 2013, Newitt *et al* 2021). Medical imaging was performed on a Siemens 1.5 T MRI scanner (TR = 5.15 ms, TE = 2.37 ms, FA = 12°, FOV = 117 × 300 × 160 mm, 1 mm isometric resolution, partial Fourier—phase/fat saturated) and was selected for this study due to the high degree of tissue heterogeneity in the breast and a longer ultrasound pathway to the tumor (see figure 2(C)).

The three MRI scans were processed with zero-filled interpolation (ZFI) (Bernstein *et al* 2001) and a homomorphic unsharp masking (HUM) filter (Guillemaud 1998). ZFI resulted in isometric ∼0.333 mm/voxel for patients 1 and 3, and ∼0.5 mm/voxel for patient 2. The HUM filter reduced artifacts in the scans from the MR bias field, and used observationally determined cutoff frequencies of 0.25, 0.17, and 0.04 cm^−1^ for patients 1, 2, and 3 respectively.

### 2.2. Segmentation

Nine researchers each segmented the three MRI scans using the software Seg3D (NIH Center for Integrative Biomedical Computing, Salt Lake City, UT, USA) (CIBC 2015). During segmentation, researchers categorized voxels into seven labels: skin, adipose tissue, fibroglandular tissue (FBGL), tumor, biopsy marker, water, and background (figure 1). The ‘background’ label contained all tissues sufficiently in the acoustic far field.

Researchers generating the segmentations had no prior experience with segmentation or medical imaging. Each researcher received approximately three hours of video training that included radiologist instruction on interpreting breast MRI, a breast segmentation demonstration, and Seg3D documentation describing built-in capabilities and tools. Researchers were also provided a training manual that described specific tools and settings in Seg3D for breast MRI segmentation. While using previously untrained researchers for segmentation means study results will not reflect true clinical interobserver variability, this study will still provide an initial assessment of the impact interobserver variability could have on MRgFUS outcomes in a SBTP framework.

### 2.3. Assessment of interobserver variability

After the segmentation datasets were created, they were compared using overlap- and distance-based metrics to quantify interobserver variability. Datasets were also used as inputs for MRgFUS treatment simulations.

#### 2.3.1. Segmentation similarity metrics

The first of two overlap-based metrics was the surface Dice similarity coefficient (sDICE) (Nikolov *et al* 2021a, 2021b). sDICE uses label surfaces rather than volumes (as is standard in the common Dice similarity coefficient (Dice 1945)) and generates a value for each labeled tissue type in a pair of segmented datasets. The method for calculating sDICE is extensive and shown elsewhere, and sDICE calculations were performed using the recommended open-source implementation (Nikolov 2021a) with a spatial tolerance factor equal to the width of 3 voxels, or ∼1–1.5 mm.

The second overlap-based metric was a multi-label, volume-weighted modification of the Dice similarity coefficient (wDICE) (Crum *et al* 2006). The volume weighting in wDICE is intended to reduce an instability of overlap metrics—such as the Dice coefficient—which tend to show greater variability for smaller structures simply because of their limited volume. wDICE measures the degree of overlap for each label then combines them into a single value using volume weighting factors unique to each label. wDICE is defined in equation (1):

\begin{equation*}{\mathrm{wDICE}} = \frac{{{{\mathop \sum \nolimits}}_{l = 1}^n2{\alpha _l}\left| {{A_l}{{\mathop \cap \nolimits}} {B_l}} \right|}}{{{{\mathop \sum \nolimits}}_{l = 1}^n{\alpha _l}\left( {\left| {{A_l}} \right| + \left| {{B_l}} \right|} \right)}}\end{equation*}

where $A$ represents one segmented dataset and $B$, a second dataset. In this context $l$ represents an individual label, $n$ is the number of labels, and ${\alpha _l}$ is a volume-weighting factor defined as:

\begin{equation*}{\alpha _l} = \frac{2}{{\left| {{A_l}} \right| + \left| {{B_l}} \right|}}.\end{equation*}

The modified Hausdorff distance (MHD), a distance-based metric, was used to account for label boundary misalignment outside of the tolerance factor used in sDICE. MHD uses an average distance *d* rather than the maximum value used in traditional Hausdorff distance (Dubuisson and Jain 1994, Taha and Hanbury 2015). Equation (3) defines MHD as:

\begin{equation*}{\mathrm{MHD}}\left( {A,B} \right) = \max \left(d\left( {A,B} \right),d\left( {B,A} \right)\right)\end{equation*}

where the average distance *d* is defined by,

\begin{equation*}d\left( {A,B} \right) = \frac{1}{{{N_a}}}\sum\limits_{a \in A} \mathop {\min }\limits_{b \in B} \parallel a - b\parallel .\end{equation*}

Here, $a$ and $b$ refer to individual geometric points in $A$ and $B$, and ${N_a}$ refers to the number of points in dataset $A$.

For each of the three patients and every potential pairing of the nine segmentation datasets (36 pairs per patient), sDICE, wDICE, and MHD were calculated. sDICE and MHD generate values for each label in each pairing. wDICE produces one value for each pairing.

### 2.4. Acoustic and thermal simulations

After segmentation datasets were completed, material properties from the literature (Duck 1990, Welsch *et al* 1993, Nam *et al* 2013, Ono 2020, Said Camilleri *et al* 2022, Baumgartner *et al* 2025) were assigned to each label as reported in table 1. The background label was assigned the same properties as water. Biopsy marker material and type were not available in patient records and so were assumed to be made of a common biopsy marker material: alpha-beta titanium alloy, Ti-6Al-4 V. For all tissues, properties were assumed constant with values selected at 37 °C when possible. When converting acoustic pressures to power deposition, tissue absorption coefficients were defined as the acoustic attenuation coefficient scaled by a constant of 0.54 to account for scattering effects (Duck 1990, Richards *et al* 2024).

**Table 1.** Tissue and material properties used in simulations.

	Speed of sound (m s^–1^)	Attenuation coefficient (Np cm^–1^*MHz)	Thermal conductivity (W m^–1^*°C)	Specific heat capacity (J kg^–1^*°C)	Blood perfusion coefficient (kg m^–3^*s)	Density (kg m^–3^)
Water	1482	0.00025	0.6	4178	0	994
Skin	1624	0.212	0.37	3391	1.9	1109
Fibroglandular	1505	0.086	0.328	3398	2.6	1066
Adipose	1440	0.044	0.171	2220	0.9	932
Tumor	1584	0.138	0.511	3610	4.8	1066
Biopsy marker	6110	0.038	6.7	565	0	4428

Acoustic simulations were performed using the hybrid angular spectrum (HAS) method to predict acoustic power deposition in the breast (Vyas and Christensen 2012). HAS was chosen for its combination of computational efficiency and ability to model heterogeneous tissues (Hansen *et al* 2021, Richards *et al* 2024).

For Patient 1 and Patient 2, transducer positioning, power, and sonication duration were defined by the MRgFUS ablation treatment. In patient 1, two 30 s, 85 W sonications were simulated. Patient 2 also had two sonications, but with shorter duration (25 s) and lower power (50 W). 10 s of post-sonication data were also simulated to capture thermal dose accumulated as the tissues cooled.

Transducer positioning for hypothetical MRgFUS Patient 3 was chosen to investigate a long, highly heterogeneous ultrasonic path within the breast. A single 30 s, 85 W sonication was simulated with a 10 s cooling period.

For all three patients, the predicted acoustic power deposition was used as an input to an explicit finite-difference time-domain (FDTD) thermal solver of the Pennes bioheat transfer equation. Previous studies have shown that the spatial resolutions used in this thermal solver are sufficient to capture the spatial and temporal peaks of the thermal distributions (Dillon *et al* 2014). Adiabatic boundary conditions were applied at the model edges and an initial condition of 37 °C was used. A temporal resolution of 0.00125 s satisfied the stability criterion inherent to explicit FDTD methods .

All acoustic and thermal simulations were performed using MATLAB version 2024a on a computer with a 3.6-GHz, i7-12 700 K core processor (Intel Corp., CA) and 32 GB of RAM.

### 2.5. Treatment outcome prediction

Predicted MRgFUS temperature distributions were used to calculate three treatment outcomes of interest, representing the magnitude of heating, focus location, and the volume of treated tissue. The magnitude of heating was represented by maximum temperature rise (Δ*T*_max_), which was defined as the largest temperature increase at any location in the breast at any time during the simulated treatment.

The thermal dose volume (TDV) was calculated from the tissue time-temperature history using the cumulative equivalent minutes a region spent at 43° C (CEM43). 240 CEM43 was used as the threshold for tissue necrosis (Sapareto and Dewey 1984, Solovchuk *et al* 2013).

The focus location was characterized using the distance between the transducer’s targeted focus location and the thermal center of mass (TCOM). TCOM was determined using a threshold value of one-half Δ*T*_max_ (Dillon *et al* 2018, Bates *et al* 2025).

### 2.6. Regression analysis

To examine the relationships between similarity metrics and simulated outcomes of interest, we conducted exploratory statistical modeling. The small sample size (*n* = 3 patients) limits the strength and generalizability of conclusions from this analysis. Nevertheless, the regression can serve as a hypothesis-generating step and illustrates a systematic modeling framework that could be applied with a more clinically representative dataset to identify which similarity metrics most influence MRgFUS simulation outcomes. This regression modeling was repeated for each of the three simulated outcomes of interest.

#### 2.6.1. Data preparation

To create a reference dataset against which each researcher segmentation dataset could be compared, a segmentation was created for each patient using the simultaneous truth and performance level estimation (STAPLE) algorithm (Warfield *et al* 2004). A binary STAPLE implementation from the open-source SimpleITK library (Yaniv *et al* 2018) was applied for each of the seven tissue-type labels. Probability maps were produced for each label, and each voxel was assigned the label with the highest probability. The resulting reference segmentations were reviewed for non-interpretive errors (e.g. skin or water labels appearing within the interior of the breast), and none were identified. These STAPLE-derived segmentations are not intended to represent ground truth or clinical reality; rather, they provide a consistent, objective baseline for internal comparison within this study.

Acoustic and thermal simulations were performed using the reference segmentation dataset for each of the five total sonications in three patients as described previously. Differences in clinically relevant outcomes (simulated maximum temperature rise, distance between geometric focus location and TCOM, and TDV) between the reference and each researcher segmentation dataset were used as the dependent variables in multiple regression models. Independent variables included sDICE, MHD, and wDICE values evaluated between each researcher and the reference segmentation dataset.

Additional modifications were implemented to prepare the data for regression modeling. *Z*-standardization was performed on all segmentation dataset comparison metrics. A binary dummy variable, ‘location’, was used to differentiate between sonication locations on the same patient. Box-cox transformations were used to normalize the distributions of the dependent variables (Box and Cox 1964, Sakia 1992).

#### 2.6.2. Exploratory modeling

Given the limited number of independent patient cases, multiple regression using the ordinary least squares method was used for exploratory, hypothesis-generating analysis. For each segmentation–sonication combination, the dependent variables were defined as differences—relative to the reference segmentations—in Δ*T*_max_, TDV, and target-to-TCOM distance. The exploratory model set included all models with one, two, or three similarity metrics as independent variables. Models with more than three metrics were excluded due to highly variable coefficient estimates.

Next, the exploratory model set was filtered for non-representativeness. All models were fit using the full dataset (three patients) to assess multicollinearity and baseline explanatory performance under the most optimistic conditions. Multicollinearity was evaluated using variance inflation factors (VIF), and models were excluded if any variable had a VIF greater than five. Although informal, this threshold is consistent with or more stringent than commonly used criteria (O’brien 2007, Vu *et al* 2015, Marcoulides and Raykov 2019).

After filtering for multicollinearity, any models with adjusted *R*^2^ $ \le 0$ were also removed. An adjusted *R*^2^ value at or below this threshold indicates that, even in this optimistic, small-sample size scenario where overfitting is likely, the model was still not capable of explaining variation in the simulated outcome of interest.

Models that passed these initial filtering steps were evaluated using leave-one-out cross-validation. Each model was trained and tested three times, once for each possible train/test split of the three patients. For convenience, these train/test splits are referred to as sub-models. Mean squared error (MSE) was calculated for each sub-model. Parent models were discarded if the maximum MSE exceeded five times the mean or median MSE across sub-models. This step helped remove models overly dependent on a single patient.

Models that passed all filtering steps were considered to have some explanatory power for variation in simulated outputs and to be free from major multicollinearity and single-patient dependence.

For these models, four summary metrics were calculated: adjusted variable frequency, sign consistency, median standardized coefficient magnitude, and the inter-quartile range of the coefficient magnitude. These metrics were used to characterize model behavior and variable stability, not to support statistical inference.

Adjusted variable frequency is defined in equation (5), where ${n_{\mathrm{initial}}}$ is the number of models within the initial exploratory model set that included the variable, and ${n_{\mathrm{final}}}$ is the number of surviving models in which it appears. A value of one indicates that all models containing the variable survived the filtering steps, while a value of zero indicates that none remained.

\begin{equation*}{\mathrm{Adjusted}}\;{\mathrm{Variable}}\;{\mathrm{Frequency}} = \frac{{{n_{{\mathrm{final}}}}}}{{{n_{{\mathrm{initial}}}}}}.\end{equation*}

Sign consistency was calculated using equation (6), where $n$ is the number of surviving models containing the variable and ${\mathrm{sgn}}\left( x \right)$ denotes the sign of the coefficient. A value of 1 indicates a consistent sign across all models, while a value of zero indicates an even split between positive and negative signs.

\begin{equation*}{\mathrm{Sign}}\,{\mathrm{Consistency}}\, = \frac{{\left| {{{\mathop \sum \nolimits}}_{i = 1}^n{\mathrm{sgn}}\left( x \right)} \right|}}{n}.\end{equation*}

Median standardized coefficient magnitude and the corresponding inter-quartile ranges were also calculated for each variable across the surviving model set.

## 3. Results

### 3.1. Segmentation datasets

**Figure 3.**
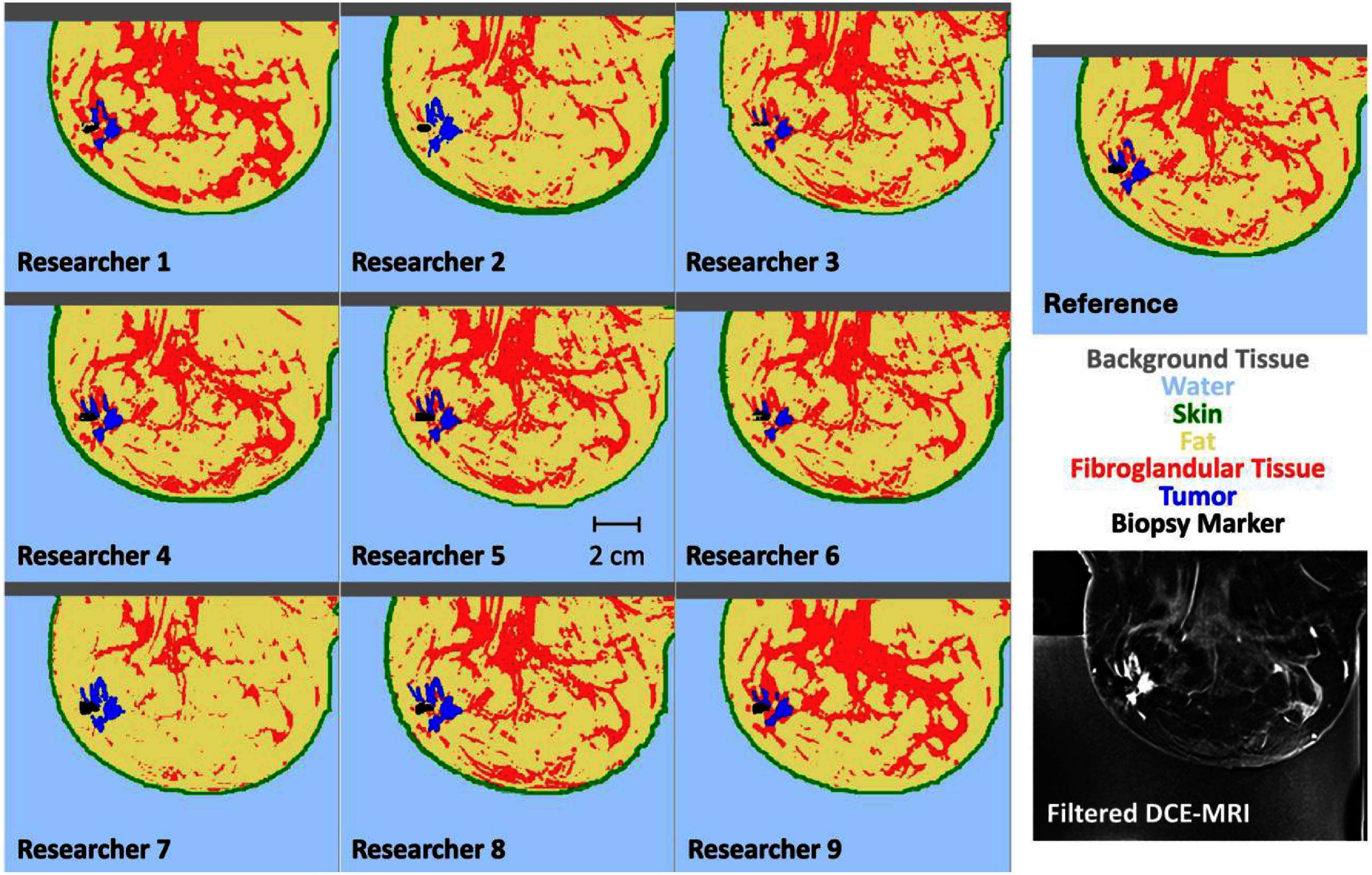
A single cross-section of the Patient 1 segmentation datasets and the corresponding filtered DCE-MRI. Datasets on the left were created by different researchers, and the top right dataset is the reference STAPLE-generated segmentation. Color coding of tissue labels is listed at right.

Figure 3 shows the nine researcher-generated segmentation datasets for Patient 1, the STAPLE-generated reference segmentation dataset, and the filtered MRI scan used for segmentation. Color coding of figure 3 labels is consistent with that of figure 1. Visually apparent differences amongst the datasets include skin thicknesses, fibroglandular-adipose tissue boundaries, and the size and shape of the tumor and biopsy marker. Similar segmentation visualizations for Patient 2 and Patient 3 are provided in the Supplementary Material.

Table 2 shows the average volumes of each label in each patient. Volumes for individual researcher and reference segmentations are provided in the Supplementary Material.

**Table 2.** Average label volumes (cm^3^) for segmentation datasets of each patient.

Patient	Background	Water	Skin	Fat	Fibroglandular tissue	Tumor	Biopsy marker
1	1280	2280	51.46	488.2	91.32	1.381	0.101
2	2579	5205	143.2	1327	205.5	1.950	0.177
3	2238	1298	49.71	412.9	68.95	3.229	0.017

### 3.2. Segmentation dataset comparison

**Figure 4.**
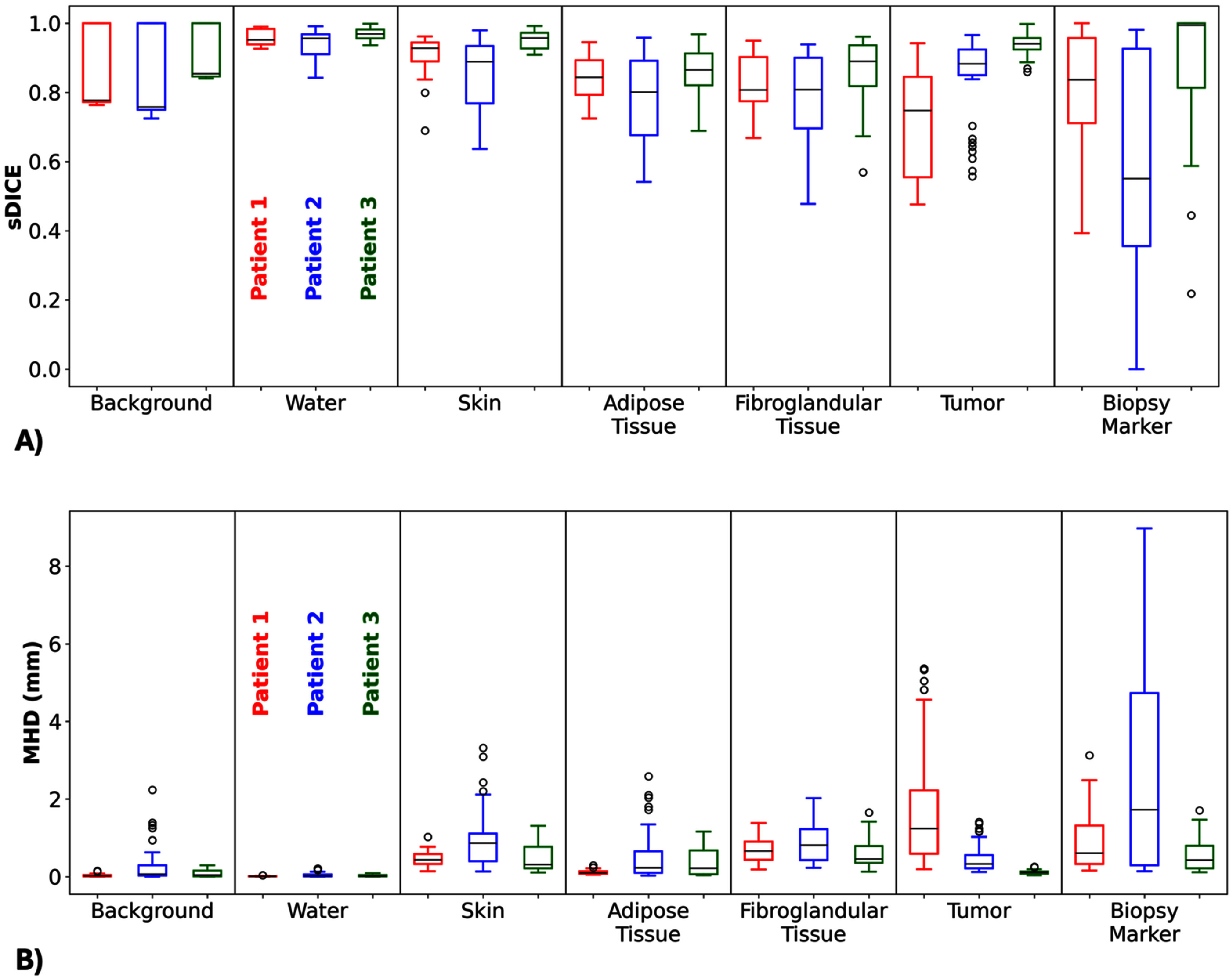
(A) Distributions of sDICE (high values are desirable) for the different tissues and patients across all 36 pairings of segmentation datasets. (B) Distributions of MHD (low values are desirable) for the different tissues and patients across all pairings of segmentation datasets. Black lines represent median values in both (A) and (B).

Figure 4(A) shows the distributions of sDICE values by label and patient. An sDICE value of one indicates perfect boundary overlap (within the three-voxel tolerance factor), and a value of zero indicates no boundary overlap. Figure 4(B) shows the MHD distributions, where values closer to zero indicate greater similarity between boundaries. Black lines in figure 4 indicate the median value with boxes covering the interquartile range and whiskers extending to the minimum and maximum, or to the furthest data points within 1.5 times the interquartile range. Outliers beyond the whiskers are shown as individual points. Generally, background and water labels have the least interobserver variability. The biopsy marker label has noticeably higher levels of interobserver variability.

Median values for the segmentation comparison metrics indicate which labels are least similar in each patient. For Patient 1, the tumor label had the lowest median sDICE value (0.747) and the highest MHD (1.236 mm). In Patient 2, the biopsy marker was the least similar (lowest median sDICE: 0.551, highest median MHD: 1.720 mm). The two metrics disagreed for Patient 3, with a median background tissue sDICE of 0.854 indicating that it was the least similar while the MHD identified the fibroglandular tissue label as the most different (highest median: 0.455 mm).

Standard deviations of the figure 4 distributions describe the range of segmentation results. In each patient, the water label had the smallest standard deviations with sDICE values of 0.023, 0.043, and 0.018 and MHD values of 0.008 mm, 0.071 mm, and 0.027 mm for Patient 1, 2, and 3, respectively. This indicates that, for the water label, the degree to which each of the nine segmentation datasets differs from the other eight is consistent. A higher label standard deviation indicates that some of the segmentation datasets differ largely from the others for that label. In the three patients observed here, the standard deviations for sDICE were always largest for the biopsy marker label (Patient 1: 0.164, Patient 2: 0.354, Patient 3: 0.180). For MHD, the biopsy marker had the highest standard deviations in Patient 2 and 3 (3.057 mm and 0.409 mm, respectively), while the tumor had the largest standard deviation (1.614 mm) for Patient 1.

The median values of figure 4 further show that Patient 3 segmentation datasets have less overall variation than datasets for Patient 1 and 2. Across all labels, median sDICE values for Patient 3 are more similar than the median values for Patients 1 and 2. The median MHD results show that background, water, and adipose tissue labels are slightly more similar in Patient 1 than in Patient 3, but for skin, fibroglandular tissue, tumor, and biopsy marker, Patient 3 is the most similar.

**Figure 5.**
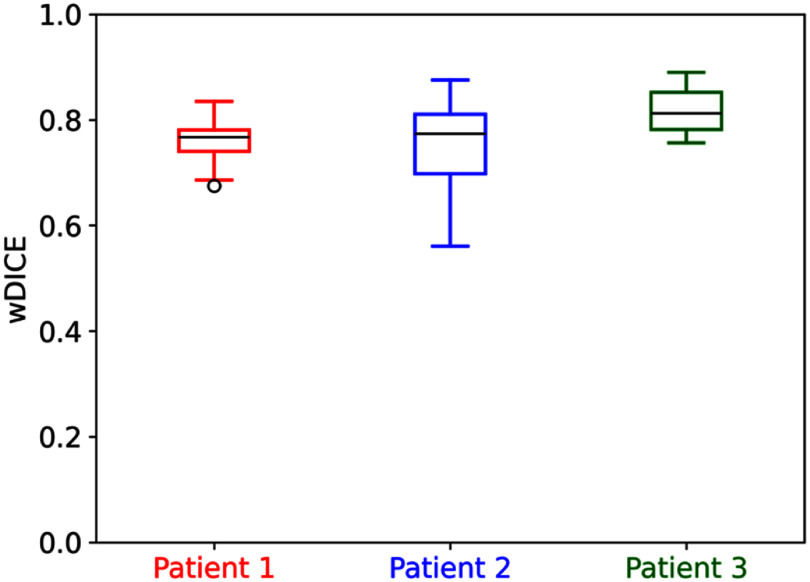
Distribution of wDICE segmentation comparisons for each patient. High values are desirable; a value of 1 indicates perfect overlap between segmentation datasets, and a value of 0 indicates no commonality. Black lines represent median values.

This observation is also reflected in the wDICE metric, which provides a convenient single-value comparison of segmentation datasets (0–1, interpreted the same as sDICE values). As shown in figure 5, Patient 3 has the highest median wDICE value of 0.812, with lower sDICE values of 0.767 and 0.774 for Patient 1 and 2, respectively.

### 3.3. Treatment outcomes

**Figure 6.**
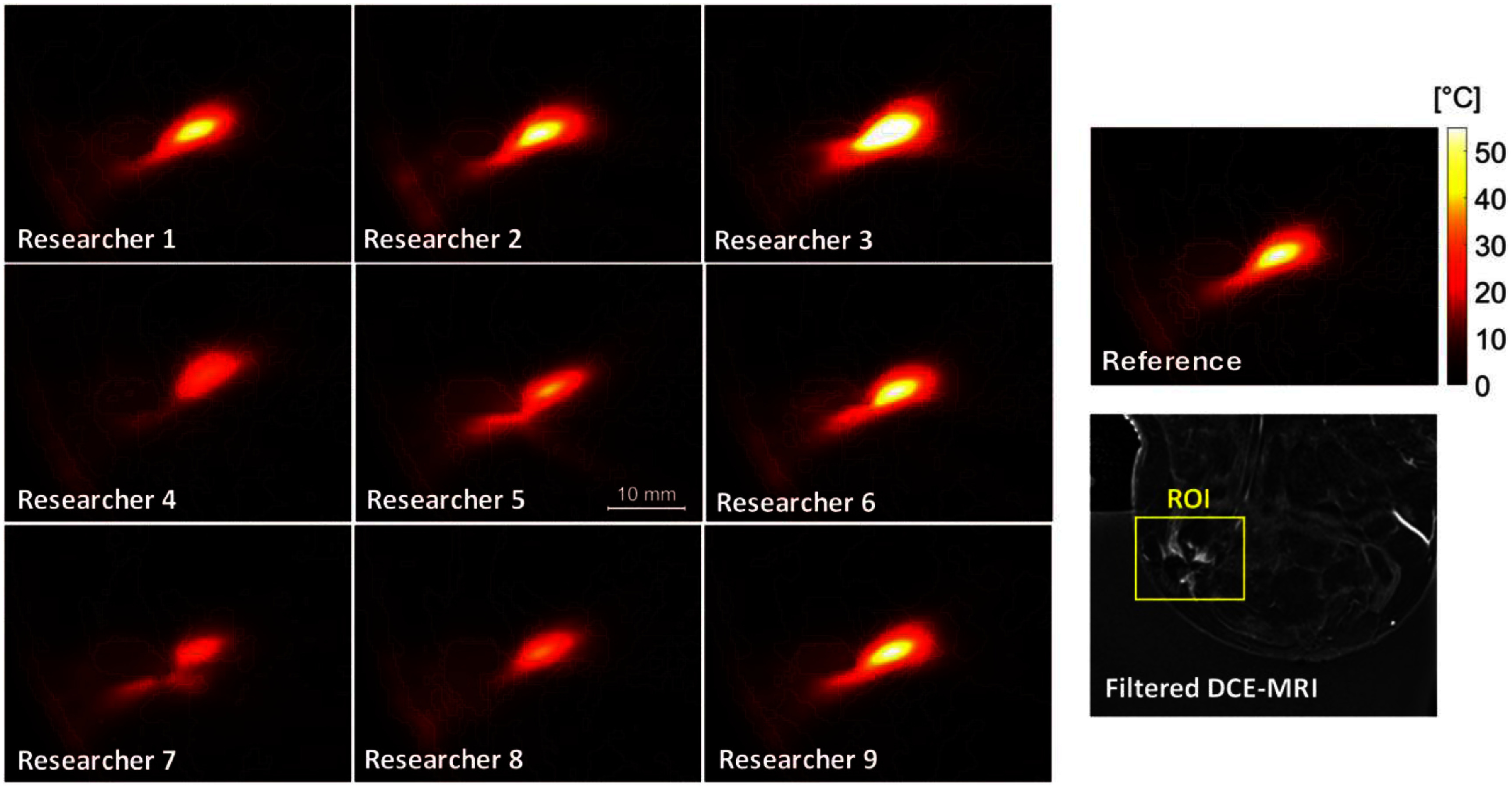
The simulated temperature rise of Patient 1, sonication 2 for each segmentation dataset in a region of interest (ROI) indicated with the yellow box in the filtered DCE-MRI scan shown in the bottom right. Gray lines in the temperature rise images indicate the segmentation boundaries of each dataset.

Figure 6 visualizes the temperature rise for Patient 1, Sonication 2 across all nine researcher-generated segmentations and the STAPLE-generated reference segmentation. The region of interest (ROI) for the temperature distributions is indicated by the yellow box in the filtered DCE-MRI image. The ultrasound transducer was located below and to the left of this image, sonicating at an oblique angle into the breast tumor (see figure 2). The displayed slice captures the location of maximum temperature in the reference segmentation. Temperature scales are consistent across images and capped at a 55 °C increase.

Temperature distributions for other patients and sonications, as well as predicted treatment outcome metrics (Δ*T*_max_, TDV, and target-to-TCOM distance), are provided in the Supplementary Material.

**Figure 7.**
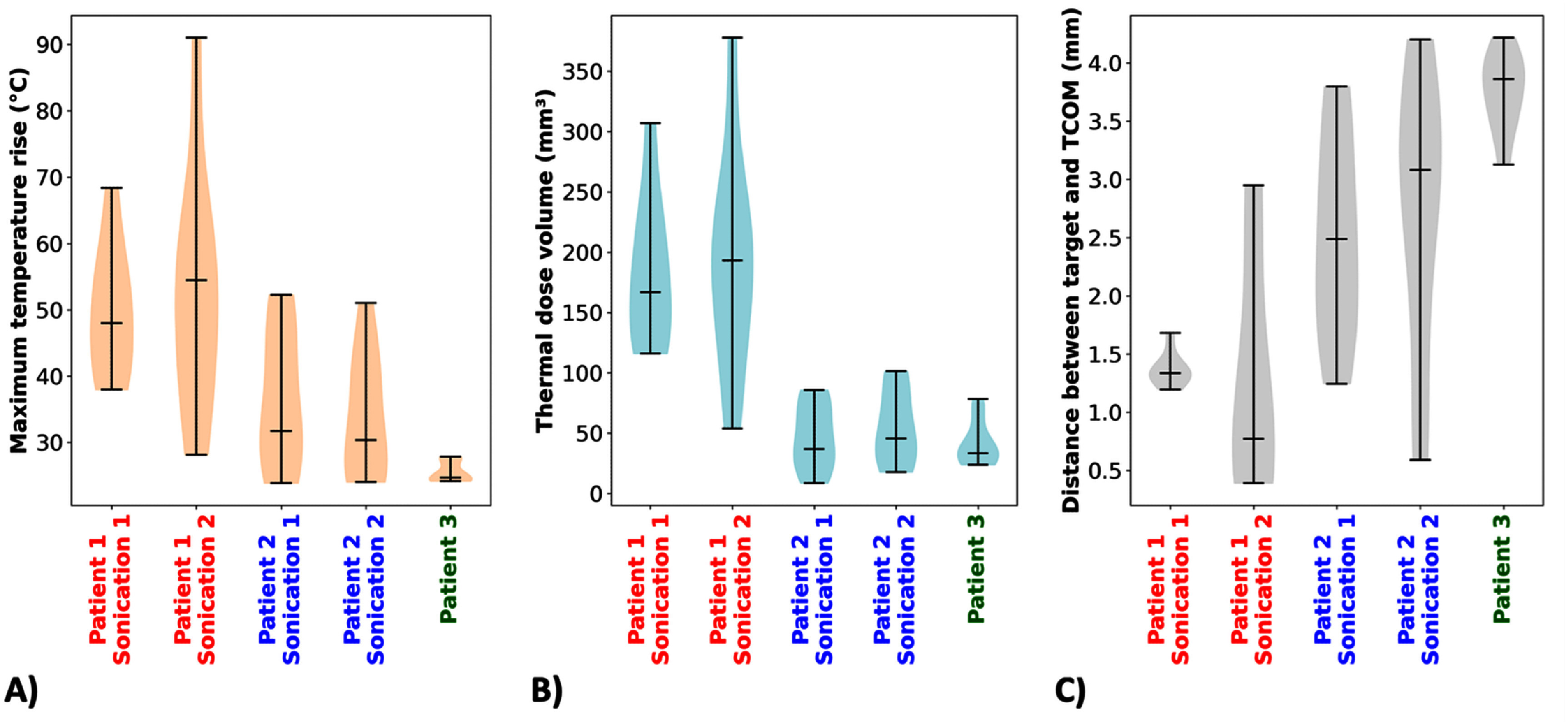
Distributions of the treatment outcome predictions for all five sonications (across three patients). Lines represent the minimum, maximum, and median values. (A) Maximum temperature rise (°C). (B) Thermal dose volume (mm^3^), which is the volume of tissue that reached 240 CEM43. (C) Distance between the geometric focus and the thermal center of mass (mm).

An overview of the treatment outcome predictions for the five simulated MRgFUS sonications in the three patients are shown in figure 7. Figure 7(A) demonstrates that the maximum temperature rise differs by as much as 62.9 °C in Patient 1, Sonication 2. The predicted TDV has differences as large as 324 mm^3^ (also in Patient 1, Sonication 2). In Sonication 2 for Patient 2, the differences between the target and the TCOM varied by as much as 3.6 mm.

Other notable patterns shown in figure 7 include the two sonications for Patient 1 having the highest median maximum temperature rise (48.0 °C and 54.5 °C for Sonication 1 and 2) and TDV (166.9 mm^3^ and 193.2 mm^3^), while also having the lowest median distances between the target focus and the TCOM (1.34 mm and 0.77 mm). Patient 3 had the opposite trend, with the lowest median maximum temperature rise and TDV of the five simulated sonications (24.7 °C and 33.3 mm^3^), along with the highest median distance between the target focus and the TCOM (3.86 mm).

### 3.4. Regression results

Three exploratory model sets were constructed, one for each simulated outcome: maximum temperature rise (Δ*T*_max_), TDV, and target-to-TCOM distance. Using fifteen different similarity metrics as inputs, each set initially contained 575 models. These models were then subjected to the regression analysis and filtering described in the Methods. After filtering, 413, 381, and 279 models remained for Δ*T*_max_, TDV, and target-to-TCOM distance, respectively.

Tables 3–5 present the adjusted variable frequency and sign consistency to indicate which of the fifteen similarity metrics best describe the segmentation differences driving outcome variability. Table 3 show results for the exploratory model set related to Δ*T*_max_, table 4 for TDV, and table 5 for target-to-TCOM distance.

**Table 3.** Regression results for the 413 surviving models related to maximum temperature rise Δ*T*_max_. Variables are ordered by descending adjusted variable frequency; this ordering does not imply greater importance than that of sign consistency.

Variable	Adjusted variable frequency	Sign consistency
wDICE	0.85	1.0
sDICE—Background	0.85	0.11
MHD—FBGL	0.82	0.47
MHD—Biopsy Marker	0.78	1.0
sDICE- Tumor	0.77	1.0
sDICE—Water	0.76	0.7
sDICE—Biopsy Marker	0.75	1.0
MHD—Tumor	0.7	1.0
sDICE—FBGL	0.68	0.64
sDICE—Skin	0.67	0.63
sDICE—Fat	0.66	0.4
MHD—Background	0.57	1.0
MHD—Water	0.56	1.0
MHD—Fat	0.56	1.0
MHD—Skin	0.55	0.69

**Table 4.** Regression results for the 381 surviving models related to TDV. Variables are ordered by descending adjusted variable frequency; this ordering does not imply greater importance than that of sign consistency.

Variable	Adjusted variable frequency	Sign consistency
wDICE	0.85	1.0
MHD—Biopsy Marker	0.79	1.0
sDICE—Tumor	0.77	0.85
sDICE—Biopsy Marker	0.75	1.0
MHD—FBGL	0.74	0.74
sDICE—Background	0.7	0.16
sDICE—FBGL	0.7	0.84
sDICE—Water	0.68	0.42
MHD—Fat	0.57	1.0
MHD—Water	0.57	1.0
MHD—Background	0.57	1.0
MHD—Tumor	0.57	0.97
sDICE—Fat	0.56	0.22
sDICE—Skin	0.53	0.07
MHD—Skin	0.51	1.0

**Table 5.** Regression results for the 279 surviving models related to the target-to-TCOM distance. Variables are ordered by descending adjusted variable frequency; this ordering does not imply greater importance than that of sign consistency.

Variable	Adjusted variable frequency	Sign consistency
wDICE	0.84	0.96
sDICE—Biopsy Marker	0.73	1.0
MHD—Biopsy Marker	0.69	1.0
sDICE—Background	0.61	1.0
sDICE—Skin	0.57	0.77
sDICE—FBGL	0.52	0.56
MHD—FBGL	0.46	0.22
sDICE- Tumor	0.45	0.33
sDICE—Water	0.41	0.3
sDICE—Fat	0.38	0.55
MHD—Fat	0.36	0.42
MHD—Background	0.35	0.19
MHD—Water	0.35	0.41
MHD—Skin	0.33	0.03
MHD—Tumor	0.21	0.64

Recall that an adjusted variable frequency of 1 indicates that all models containing the variable survived filtering, while 0 indicates that none did. For sign consistency, a value of 1 indicates a consistent coefficient direction (positive or negative), whereas 0 indicates an even split, suggesting instability. Thus, similarity metrics with values closer to 1 in both columns of tables 3–5 could be reliable descriptors of segmentation variability driving the observed variability in Δ*T*_max_, TDV, and target-to-TCOM distance, respectively.

Because many of the similarity metrics are label-specific, tables 3–5 also show that variability may be driven more by certain tissue labels than others. Notably, wDICE and biopsy marker-related metrics appear frequently and with consistent effect direction across all three outcomes. Tumor-related metrics show similar patterns for models related to the maximum temperature rise and TDV.

Median coefficients (standardized across metrics) and inter-quartile ranges from the surviving datasets were also calculated and are provided in the Supplementary Material. Although these metrics may be sensitive to overfitting given the small sample size, they were consistent with the regression results for adjusted variable frequency and sign consistency: wDICE and biopsy marker–related metrics exhibited larger median coefficients and tighter interquartile ranges than other metrics.

## 4. Discussion

SBTP platforms for MRgFUS will likely incorporate automated tissue segmentation using deep learning, as manual segmentation by experts is too time-intensive for routine clinical use. Medical image segmentation tools have shown rapid improvement and can achieve near expert-level performance (Kaiser *et al* 2019, Wang *et al* 2021, Webb *et al* 2021). However, interobserver variability in training data contributes to uncertainty in automated outputs (Jungo *et al* 2018, Yu *et al* 2020). Ongoing work aims to better characterize these effects and develop models that account for such variability (Jungo *et al* 2018, Wang *et al* 2021, Athanasiou *et al* 2023).

Another valuable perspective is understanding how interobserver variability in segmentation affects downstream outcomes for specific segmentation applications (Vinod *et al* 2016a). Prior work has examined this sensitivity in CT-based segmentation for radiotherapy dose calculations (Covert *et al* 2022) and breast MRI tumor segmentation for radiomics (Granzier *et al* 2020).

The work presented here provides a foundation for similar analysis of how interobserver variability in full breast MRI segmentation influences simulated MRgFUS treatment outcomes. This approach could inform segmentation guidelines that ensure simulations better reflect reality and support the development of well-validated datasets for training automated deep learning algorithms.

Prior to this study, interobserver variability had not been examined in the context of MRgFUS segmentation. We hypothesized that if simulated MRgFUS outcomes were insensitive to variability in our non-expert dataset, interobserver variability could be largely dismissed as a concern. However, the results of our exploratory study show that simulated outcomes are sensitive to interobserver variability in segmentation, indicating that it warrants further investigation.

The remainder of the Discussion section details the analyses supporting this conclusion, the rationale for our similarity metric selection, and a proposed roadmap for defining acceptable, quantitatively described levels of variability in datasets used to train MRgFUS segmentation models.

### 4.1. MRgFUS outcome sensitivity

The key finding of this study was that interobserver variability in breast cancer segmentation datasets had meaningful impacts on simulated MRgFUS treatment outcomes. As shown in figure 7, differences in maximum temperature rise predictions were as large as 62.9 °C and differences between TDV predictions for a single sonication were as large as 324 mm^3^. These differences indicate that MRgFUS simulations were highly sensitive to our non-expert interobserver variability in segmentation. Further study to determine whether variability in expert datasets is sufficiently low for SBTP is warranted.

Observed patient-specific patterns were consistent with acoustic principles. Patient 3 exhibited the lowest maximum temperature rise and the largest target-to-TCOM distance, likely due to a deeper tumor compared to Patients 1 and 2. Increased depth introduces more tissue and interfaces along the acoustic path, leading to greater scattering, phase aberration, and attenuation. These effects reduce targeting precision (larger target-to-TCOM distance) and energy deposition (lower maximum temperature rise).

### 4.2. Similarity metric selection and observations

Segmentation serves many purposes, and evaluation metrics should reflect the intended application; however, this is sometimes overlooked. The Dice similarity coefficient is frequently used as a default metric, despite not being appropriate for all tasks (Taha and Hanbury 2015, Kofler *et al* 2023, Ma *et al* 2024, Maier-Hein *et al* 2024). First, it is inherently sensitive to label size. A single-voxel difference has a much larger impact on small structures than on large ones. It also measures only voxel-wise overlap, making it insensitive to orientation shifts or boundary offsets and less suitable for complex shapes. As shown in figure 3 and table 2, multi-tissue breast MRI segmentation involves both complex geometries and labels spanning multiple orders of magnitude in volume, making the Dice similarity coefficient inappropriate for this application.

For this study, metrics were selected for their ability to represent differences in label boundaries and in label volumes (Taha and Hanbury 2015). Two overlap-based metrics—the surface Dice coefficient (sDICE) and the weighted Dice coefficient (wDICE)—and one distance-based metric, the MHD were used. Each metric addresses different limitations of the commonly used Dice similarity coefficient. sDICE is based on surface overlap rather than full volume overlap, reducing the bias of Dice against small-volume labels with large surface areas. It also incorporates a tolerance factor, which enables sDICE to account for a limited degree of boundary complexity and alignment differences. wDICE addresses Dice’s sensitivity to label size and its limitations in multiclass settings by computing a volume-weighted average across labels. This yields a single, interpretable score per segmentation pair, which may be useful for defining domain-specific standards.

Unlike sDICE and wDICE, MHD is a distance-based metric that captures boundary alignment differences beyond the sDICE tolerance. Distance-based metrics quantify boundary discrepancies directly but can be sensitive to outliers. MHD was selected for this study because it accounts for that sensitivity using an average distance (see equation (4)), rather than a maximum (as in standard Hausdorff distance).

These metrics were selected to account for the complex shapes and varying label volumes in MRgFUS segmentation. In particular, sDICE and MHD provide more detailed insight into where interobserver variability occurs. In this study, large variability in biopsy marker MHD and sDICE values indicates substantial segmentation differences for that label. Notably, the label with the greatest variability differed by patient, suggesting a role for patient-specific anatomy.

Patient 3 showed the lowest interobserver variability among the three patients (see figures 4 and 5). Possible explanations include differences in MRI acquisition (equipment and parameters), clearer or more regular anatomy, or increased researcher experience, as Patient 3 was segmented last by all researchers. The smaller biopsy marker volume in Patient 3 (see table 2) and its greater distance from the target may have contributed to reduced interobserver variability.

Given the small dataset of this exploratory study, observed trends are not generalizable. Rather, this work demonstrates how the similarity metrics and regression analysis improve our ability to interpret segmentation differences, with results intended to support hypothesis generation for future studies.

### 4.3. Regression analysis

The level of variation in the simulated outcomes indicates that simulations are sensitive to interobserver variability. The segmentation comparison metrics help to explain in which labels interobserver variability takes place. The regression analysis performed sought to connect all of these variables, and to explain which metrics best describe where interobserver variability is most influential for simulated MRgFUS outcomes.

The observed variation in simulated MRgFUS outcomes (figure 7) indicates sensitivity to interobserver variability. The similarity metrics identify which labels contribute most to interobserver variability (figures 4 and 5). The regression analysis (tables 3–5) links the similarity metrics to their influence on MRgFUS outcomes.

It is again important to note that the study’s small sample size prevents the specific conclusions drawn from this regression analysis to be generalizable. Instead, this work aims to generate hypotheses about which metrics may inform MRgFUS segmentation guidelines and to demonstrate how a regression-based approach can identify labels where reducing interobserver variability would be most impactful. Accordingly, the analysis emphasizes variable stability rather than effect size.

Among all similarity metrics, wDICE had the highest adjusted variable frequency and a consistently stable sign across all outcomes, suggesting it may serve as a robust first-line measure of overall segmentation similarity in MRgFUS, especially given its more global scope compared to the other similarity metrics.

Across all three patients, biopsy marker–related metrics were among the most stable variables in all three surviving model sets (see tables 3–5), identifying this label as one where reducing interobserver variability in segmentation would help variability in simulated outcomes. Domain knowledge supports this: the biopsy marker’s distinct material properties (table 1), combined with uncertainty in its location, shape, and composition, can strongly influence acoustic and thermal responses. These effects are evident in the temperature distributions (see figure 6 and supplementary material), where a biopsy marker in the beam path can deflect, block, or shadow the focus. The closer the biopsy marker is to the target location, the more pronounced these effects.

High variability in biopsy marker segmentations likely reflects biopsy marker-induced MRI artifacts from magnetic field inhomogeneity (Puesken *et al* 2021) and limited information about marker type and material. Incorporating detailed biopsy marker metadata and complementary imaging (e.g. mammography) could reduce this variability in future studies.

Regardless of whether the biopsy marker label is broadly impactful on MRgFUS simulation outcomes, this study demonstrates how a data-driven regression analysis can identify influential labels and guide targeted strategies to reduce interobserver variability.

### 4.4. Future work

Use of segmentation or target delineation to support predictive radiation dose calculation is an established component of treatment planning for radiotherapies (Nijkamp *et al* 2012, Ciardo *et al* 2017). Its adoption has improved treatment accuracy (Poortmans *et al* 2013, Ciardo *et al* 2017). As segmentation became more important, interobserver variability was shown to affect dosimetry calculations in ways similar to how interobserver variability influenced the treatment outcome predictions in this study (Jameson *et al* 2014, Vinod *et al* 2016a, Bell *et al* 2020). Studies have shown that establishment of expert guidelines, incorporation of atlas-based segmentation, and increased training can reduce interobserver variability (Vinod *et al* 2016b, Patrick *et al* 2021).

Because our exploratory results indicate that simulated MRgFUS outcomes are sensitive to interobserver variability and similar effects are known to impact radiotherapy, we believe further investigation in this context is warranted for MRgFUS SBTP. This study also introduces tools that may support such efforts.

Future work should focus on developing a comprehensive dataset of expert-level breast MRI segmentations. This dataset should capture diverse anatomies, tumor locations, biopsy marker types, and tissue compositions, and be created by experienced radiologists and segmentation experts with varied training backgrounds to better characterize the true clinical interobserver variability. Such a comprehensive dataset would also support the training of deep learning-based segmentation algorithms.

This study highlights wDICE, sDICE and MHD as well-suited for analyzing this expert dataset. wDICE provides an easily interpretable metric for overall differences across labels, while sDICE and MHD help identify specific labels with high variability. A regression-based approach, as demonstrated here, can further pinpoint where interobserver variability most influences simulated outcomes.

With a comprehensive expert dataset, regression-based analyses could yield generalizable conclusions to support the development of quantitative segmentation standards. Such standards would reduce interobserver variability and result in more consistent, accurate MRgFUS treatment simulations. They could also provide a data-driven quality benchmark for training deep learning segmentation algorithms, reducing reliance on expert opinion, which is difficult to verify and inherently variable (Athanasiou *et al* 2023).

### 4.5. Study Limitations

While the results indicate that simulated outcomes are sensitive to interobserver variability in segmentation, they must be interpreted in the context of several limitations.

A primary limitation is the small sample size (three patients, five sonications), which cannot represent the full range of breast anatomies. However, the goal of this study was to establish whether sensitivity in segmentation interobserver variability exists. The observed variation, even in this limited dataset, provides evidence that a comprehensive expert dataset is needed—an investment that may yield meaningful improvements in MRgFUS treatment planning. Notably, Patients 1 and 2 were participants in a MRgFUS clinical trial, suggesting that sensitivity to interobserver variability may also exist within anatomies eligible for treatment.

Another limitation is the use of non-expert segmentations, meaning results may not reflect clinical interobserver variability. This was partially mitigated through expert-led training, which we consider sufficient for demonstrating the need for future expert involvement.

The use of non-expert segmentations alongside a STAPLE-derived reference may raise concerns, particularly given STAPLE’s common use as a proxy for ground truth. Here, however, STAPLE was used solely to generate a consistent, objective baseline—not a clinical standard. The reference segmentations were also reviewed for obvious errors to ensure they met the same baseline quality as the researcher-generated segmentations.

Intra-observer variability was not explored in this study. This does not impact the core finding of the study (that differences in segmentation lead to meaningful differences in simulated treatments) but further work would be necessary to quantify differences due to intra-observer variability and propose solutions.

While this study addresses the impact of interobserver variability on predicted MRgFUS treatment outcomes, it does not characterize the accuracy of the segmentation datasets or of the predictions made from those datasets. Clinical MR temperature data from the first two patients could have been used for comparison, but the high proportion of fat in the breast greatly diminishes confidence in the quantitative accuracy of the MR thermometry (Josset *et al* 2025).

Finally, only one type of MRI contrast, dynamic contrast enhanced T1-weighted images, was used to generate the segmentation datasets. Other contrasts, including T2-weighted, diffusion weighted imaging, and potentially other contrasts contain distinct information regarding tissue boundaries and could be used alone or in combination to inform the segmentation process.

## 5. Conclusion

This exploratory study shows that interobserver variability in segmentation can lead to large differences in simulated MRgFUS breast cancer treatment outcomes. Additional work is needed to assess and potentially reduce clinical interobserver variability for SBTP to be clinically useful. While the three MRI scans used in this study are not representative of all breast cancer patient anatomies, the results identified wDICE, sDICE, and MHD as useful metrics for characterizing interobserver variability in the context of MRgFUS.

Future work should focus on developing a comprehensive expert segmentation dataset and evaluating whether metrics such as wDICE, and labels such as the biopsy marker, remain influential. This would support the development of expert segmentation guidelines that reduce interobserver variability for MRgFUS SBTP, similar to advances achieved in radiotherapy treatment planning.

## Data Availability

All data that support the findings of this study are included within the article (and any supplementary information files). Supplementary Segmentation available at https://doi.org/10.1088/1361-6560/ae7cd0/data1.
